# Combining Clinical and Epidemiologic Features for Early Recognition of SARS

**DOI:** 10.3201/eid1002.030741

**Published:** 2004-02

**Authors:** John A. Jernigan, Donald E. Low, Rita F. Helfand

**Affiliations:** * Centers for Disease Control and Prevention, Atlanta, Georgia, USA; † University of Toronto, Ontario, Canada

**Keywords:** SARS, Coronavirus, diagnosis, epidemiology, clinical features, SARS-CoV

## Abstract

Early recognition and rapid initiation of infection control precautions are currently the most important strategies for controlling severe acute respiratory syndrome (SARS). No rapid diagnostic tests currently exist that can rule out SARS among patients with febrile respiratory illnesses. Clinical features alone cannot with certainty distinguish SARS from other respiratory illnesses rapidly enough to inform early management decisions. A balanced approach to screening that allows early recognition of SARS without unnecessary isolation of patients with other respiratory illnesses will require clinicians not only to look for suggestive clinical features but also to routinely seek epidemiologic clues suggestive of SARS coronavirus exposure. Key epidemiologic risk factors include 1) exposure to settings where SARS activity is suspected or documented, or 2) in the absence of such exposure, epidemiologic linkage to other persons with pneumonia (i.e., pneumonia clusters), or 3) exposure to healthcare settings. When combined with clinical findings, these epidemiologic features provide a possible strategic framework for early recognition of SARS.

In November 2002, clusters of a highly transmissible and severe atypical pneumonia began appearing among residents of the Guangdong Province of China. These patients are now believed to have been the first persons with severe acute respiratory syndrome (SARS), a previously undescribed respiratory illness now known to be caused by a novel coronavirus (SARS-CoV) (*1*–*4*). These original clusters marked the beginning of an outbreak that spread rapidly around the globe, resulting in 8,422 reported cases from 32 countries and a case-fatality rate of 11% (*5*). On July 5, 2003, the World Health Organization (WHO) announced that all known person-to-person transmission of SARS-CoV had ceased (*6*). The cause for the decline in cases is not yet fully understood, but SARS-CoV may still possibly exist within either an animal or a human reservoir and cause future outbreaks (*4*). Clinicians and public health agencies must be prepared for the possible reappearance of SARS.

Although many unanswered questions remain regarding the epidemiology of SARS, simple infection control measures can dramatically reduce transmission of SARS-CoV (*7*–*10*). In every region in which major outbreaks were reported, a substantial proportion of cases resulted from delays in clinical recognition and isolation of SARS patients after they were admitted into the healthcare system (8*,**9**,**11**–**13*). Studies of transmission in Hong Kong, Singapore, and Ontario, Canada, suggest that early case detection will be a critical component in controlling future outbreaks of SARS (*10*,*14*–*16*).

Currently, no rapid diagnostic tests are widely available to rule out SARS. Because the early clinical features can be similar to those of other bacterial and viral infections, rapid recognition of SARS patients is likely to be particularly challenging in the context of seasonal outbreaks of other respiratory illnesses. The need for distinguishing patients with SARS from those with more common and benign illnesses presents clinicians with a diagnostic dilemma; screening methods that are not sufficiently sensitive may result in delays in recognition and uncontrolled transmission of SARS, while nonspecific screening methods could result in unnecessary isolation of large numbers of persons, rapidly overburdening the already limited resources of both the healthcare and public health systems.

A balanced approach to early recognition of SARS will require clinicians to look not only for suggestive clinical features but also for epidemiologic clues that suggest SARS-CoV infection. We provide a possible framework that combines epidemiologic features and clinical findings to formulate strategies for early recognition of SARS.

## Clinical Description of SARS

### Clinical Signs and Symptoms

The median incubation period for SARS appears to be approximately 4–6 days; most patients become ill within 2 to 10 days after exposure (*8*,*12*,*17*,*18*). Some evidence suggests that the incubation period may be as long as 14 days in some persons (*17*).

The most common initial symptom is fever, often accompanied by headache, myalgia, malaise, chills, and rigor (*1*,*11*,*17*–*22*). In some patients, headache, myalgia, and malaise precede the onset of fever by up to 1 day, and fever may have resolved by the time respiratory symptoms appear (*1*,*18*,*19*,*22*). Respiratory symptoms typically do not begin until 2–7 days after illness onset, although they are among the initial symptoms in up to 30% of patients (*1*,*18*–*20*). The most common respiratory complaints are lower respiratory tract symptoms, including nonproductive cough and dyspnea; productive cough is reported in up to 25% of patients (*1*,*11*,*17*–*22*). In some series, <10% of patients reported upper respiratory complaints (*18*,*20*,*23*), but in others the reported prevalence of rhinorrhea or sore throat is as high as 25% among patients with SARS (*11*,*17*,*19*). The prevalence of gastrointestinal symptoms has varied by report, but nausea, vomiting, diarrhea, or a combination of these symptoms has been reported in up to 25% of patients with SARS at the time of initial evaluation (*1*,*11*,*17*–*22*,*24*). In one series, diarrhea developed in 73% of patients at some point in the course of illness (*22*). Fever and diarrhea have been the dominant initial symptoms in some patients (*13*). Asymptomatic infection with SARS-CoV appears to be uncommon (*25*,*26*).

Elderly patients and those with underlying chronic illnesses such as renal failure may not have typical symptoms of SARS (*12*,*13*,*27*). For patients in this group who have strong epidemiologic risk factors for SARS, the diagnosis should be considered in almost any change in health status, even if the patients do not exhibit typical clinical features.

### Physical Findings

Tachypnea, tachycardia, and hypoxemia have been reported in 40% to 75% of patients upon admission to the hospital (*1*,*18*–*20*) but may be less common in patients who are evaluated earlier in the course of illness as outpatients (*21*). Upon auscultation of the lungs, rales or rhonchi have been detectable in most patients in some series, and less commonly in others (*18*,*20*,*21*,*28*). Some researchers have observed a lack of lung sounds despite marked infiltration on chest radiography (*21*,*28*). As many as 15%–44% of patients may have a normal measured body temperature when first evaluated (*18*,*19*).

### Laboratory Findings

Hematologic abnormalities are among the most consistent laboratory findings reported in patients with SARS; most patients have total leukocyte counts that are normal or slightly low, and 70%–95% of patients have lymphopenia (*11*,*18*–*20*,*22*,*29*). Platelet counts are mildly depressed in 30% to 50% of patients (*11*,*19*,*22*,*29*). Prolongation of the activated partial thromboplastin time can be observed in 40% to 60% of patients, but disseminated intravascular coagulation is uncommon (*11*,*29*).

Other common abnormal laboratory findings include elevated lactate dehydrogenase levels in 70% to 90% of patients (*11*,*18*,*19*), elevated alanine aminotransferase or aspartate aminotransferase levels in 20% to 30% (*11*,*22*), elevated creatine phosphokinase in 30% to 40% (*11*,*22*,*30*), and elevated C-reactive protein (*1*,*31*).

### Radiographic Findings

While the full understanding of the spectrum of radiographic manifestations of SARS will require additional information, available data suggest that almost all reported patients with laboratory evidence of SARS-CoV infection have radiographic evidence of pneumonia documented at some point during their illness (*19*,*21*,*31*). Chest radiographs may be normal in up to 30% of patients with the clinical diagnosis of SARS at the time when first evaluated (*18*,*22*,*32*–*34*). In reports from China, radiographic changes consistent with pneumonia were detected in 67% to 80% of SARS patients by day 3 of illness, 97%-100% by day 7, and in 100% by day 10 (*33*,*34*). A lesion typically begins as an isolated focal lesion found in a peripheral location, often in the lower lobes. In 75% of patients, the lesions progress over several days to involve additional lobes or both lungs (*32*,*34*).

Computed tomography (CT) of the chest appears to be more sensitive than conventional chest radiography for detecting pneumonia; SARS patients who have normal chest radiographic findings early in their clinical course often have evidence of pneumonia by CT (*11*,*35*). Common CT findings are ground-glass opacification and a lower lobe and peripheral distribution (*35*).

### Distinguishing SARS from Other Illnesses

As with other causes of bacterial and viral pneumonia, clinical findings in patients with SARS cannot accurately predict the causative agent. Further study is required to determine whether a constellation of clinical findings alone can be used to discriminate accurately between SARS and other (especially viral) respiratory illnesses. Many of the clinical and laboratory features of SARS are similar to those in other forms of viral pneumonia (*28*,*36*). Several clinical features, however, may be helpful in facilitating recognition of patients with SARS (Table 1).

**Table 1:** Common clinical features of severe acute respiratory syndrome (SARS)

Clinical feature	Common findings with SARS-associated coronavirus infection
Initial symptoms	Nonrespiratory prodrome lasting 2–7 days characterized by one or more of the following: Fever Rigors Headache Malaise Myalgia Diarrhea Respiratory phase beginning 2–7 days after onset characterized by: Nonproductive cough Dyspnea Absence of upper respiratory symptoms
Laboratory	Normal or low total leukocyte cell count
	Lymphopenia
	Mildly depressed platelet count
	Elevated lactate dehydrogenase levels
	Elevated creatine phosphokinase levels
	Elevated transaminase levels
	Prolonged activated partial thromboplastin time
Radiographic	Abnormal chest x-ray in almost all patients by the second week of illness

## Laboratory Tests for SARS-CoV

The main laboratory tests available to diagnose SARS-CoV infection are RNA detection through reverse transcriptase-polymerase chain reaction (RT-PCR) or real-time PCR and serologic testing for antibodies against SARS-CoV (*1*,*2*,*22*). None of these tests can be used reliably to detect the presence or absence of SARS-CoV infection at the time of initial evaluation. RT-PCR and real-time PCR are insufficiently sensitive to reliably diagnose all persons with SARS when first evaluated; in one study, SARS-CoV was initially detected in nasopharyngeal samples by RT-PCR in 32% of patients and in 68% at day 14 after illness onset (*22*). PCR tests also can provide false-positive test results even in the most experienced laboratories, so their indiscriminant use for persons at low risk for SARS infection could result in a false diagnosis of SARS and unnecessarily initiating isolation and quarantine measures. Although antibodies can be detected in serologic assays starting at 10 to 14 days after illness onset (*2*,*22*), serologic tests cannot reliably rule out SARS-CoV infections until 28 days after onset of symptoms, when sensitivity is at least 93% (*22*).

While respiratory samples have been the most commonly used samples for virus detection, virus may be more readily detectable in serum earlier in the course of illness and in stool samples later in the course of illness (*1*,*22*) (Centers for Disease Control and Prevention [CDC], unpub. data). More research is needed to determine the optimal timing of sample collection, the duration of shedding, and the optimal type of sample.

## Epidemiologic Features Important for Early Recognition of SARS

Given that no specific clinical or laboratory findings can with certainty distinguish SARS from other respiratory illnesses rapidly enough to inform early management decisions, epidemiologic features are critical to early recognition of SARS. Epidemiologic features that may be helpful in early recognition include a history of exposure to known SARS case-patients or SARS-affected areas, an epidemiologic linkage to a cluster of pneumonia cases, a history of travel to previously SARS-affected areas, and employment as a healthcare worker with direct patient care.

### Epidemiologic Linkage to Cases or SARS-affected Areas

The predominant mode of transmission of SARS-CoV appears to be through large respiratory droplets or direct contact (*7*,*8*). This mode of transmission is consistent with the observation that most patients can be linked, either directly or indirectly, to persons with SARS or places where transmission is either suspected or documented (*17*,*37*). In the Toronto and Singapore outbreaks, >94% of cases had documented contact with a SARS patient or with a hospital ward where there was a known SARS patient (*8*,*38*). Therefore, determining if persons with symptoms compatible with SARS have an epidemiologic linkage either to other persons with known or suspected SARS or to places with known or suspected transmission of SARS-CoV is important.

Whether a history of travel to areas previously affected by SARS will be a useful epidemiologic clue for recognizing future outbreaks depends in part on whether SARS-CoV currently exists within a human or an animal reservoir. If the virus exists within a human reservoir, the virus could reemerge anywhere on the globe, although the areas of highest activity during the recent outbreaks are most likely to harbor persistent infection in humans. Alternatively, if SARS-CoV currently exists primarily within the animal reservoir from which it originated, future outbreaks may more likely originate in Southeast Asia. Given that China appears to have been the origin of the most recent outbreak (*4*,*39*) and neighboring areas are at greatest risk, persons traveling in Southeast Asia, especially in China, Hong Kong, and Taiwan, may be at increased risk for infection if SARS recurs.

### Case Clustering

The major limitation of relying on linkage to settings of known transmission to identify persons at risk for SARS is identifying the first cases acquired in an area not previously known to have circulation of SARS-CoV. Because SARS-CoV infections tend to appear in clusters, one potential strategy for early recognition in such areas is to seek evidence for clustering of pneumonia cases. Early recognition of clusters requires clinicians evaluating patients with pneumonia to routinely seek a history of exposure to others with pneumonia.

### Healthcare Association

Healthcare facilities have played a central role in the epidemiology of SARS. Persons who work in healthcare settings were among the earliest and most severely affected group in almost every major outbreak reported, particularly during the earliest phases of the outbreak (*8*,*11*,*13*). For example, in the Toronto and Singapore outbreaks, 43% and 41%, respectively, of the SARS cases occurred in healthcare workers (*40*). Therefore, atypical pneumonia among healthcare workers should raise the suspicion for SARS, particularly if there are multiple cases among healthcare workers in the same facility. In addition, atypical pneumonia in a person who works in a laboratory that contains live SARS-CoV should raise the possibility of SARS.

## Combining Clinical and Epidemiologic Features

Since patients may transmit the virus early in the clinical course (*8*), the goal of diagnostic strategies should be to detect patients with SARS as early in the illness as possible to prevent potential transmission. A practical approach to evaluating patients with fever or respiratory symptoms is needed, which requires an assessment of the strength of the evidence of exposure to other SARS-CoV–infected persons. This assessment is directly related to the level of documented SARS activity in the surrounding community and the world.

## Evaluating Patients in the Absence of Documented SARS Activity Anywhere in the World

In the absence of any documented SARS transmission worldwide, the overall likelihood that a given patient has SARS-CoV infection will be exceedingly low unless there are both typical clinical findings and some accompanying epidemiologic evidence for SARS-CoV infection. Therefore, one approach would be to consider the diagnosis only among patients with both 1) unexplained severe pneumonia and 2) epidemiologic evidence that could suggest SARS, including a link to a cluster of cases of unexplained pneumonia, a history of recent travel (or close contact to an ill traveler) to a previously SARS-affected area, or employment as a healthcare worker with direct patient care responsibilities (Table 2). For persons who are healthcare workers or who have traveled to previously SARS-affected areas, evidence of clustered pneumonia cases would further increase the index of suspicion.

**Table 2:** Combination of clinical and epidemiologic factors that raise suspicion for SARS among patients with community-acquired illness^a^

Level of worldwide SARS activity	Clinical features	Epidemiologic features
No documented SARS activity		
	Patients with severe pneumonia of unknown cause	Recent exposure to other persons with unexplained pneumonia Recent travel to previously SARS-affected area or close contact with ill persons with a history of travel to such areas^b^ Healthcare worker^c^
SARS activity documented		
	All patients with fever, especially accompanied by headache, myalgias, rigor Any patient with lower respiratory tract symptoms	Close contact with a person with known or suspected SARS Exposure to any place in which active transmission of SARS is documented or suspected
	Patients with severe pneumonia of unknown cause	Close contact with a person with known or suspected SARS Exposure to any place in which active transmission of SARS is documented or suspected If none of the above: Recent exposure to other persons with unexplained pneumonia Recent travel to previously SARS-affected area or close contact with ill persons with a history of travel to such areas Healthcare worker

^a^The possibility of severe acute respiratory syndrome (SARS) should be considered for any patient with both the clinical and epidemiologic features described, depending upon the level of worldwide SARS activity. Final decisions on the need for SARS isolation precautions or testing for SARS-associated coronavirus infection should be made in conjunction with local health authorities. Examples of epidemiologic factors that may raise a higher index of suspicion for SARS, even in the absence of known SARS activity, include clusters of pneumonia among healthcare workers, or exposure to persons with pneumonia while traveling in a previously SARS-affected area. ^b^Previously SARS-affected areas include areas in Southeast Asia in which SARS may originate and neighboring areas that may be at risk for early spread because of importations, including China, Hong Kong, and Taiwan. ^c^Healthcare worker defined as one who has direct patient care responsibilities.

In the absence of pneumonia, history of travel to a previously SARS-affected area is likely to have an extremely low positive predictive value for detecting SARS among patients with respiratory illness and, if used as a screening tool, would likely result in an unacceptable burden on the public health system. (U.S. travelers alone make almost 5 million trips to Asia every year, and respiratory symptoms are common among returning travelers [*41**,**42*].)

Clinicians practicing within previously SARS-affected areas may have to adopt a different approach to detecting SARS among patients with pneumonia, such as requiring both evidence of clustering and a typical combination of laboratory and radiologic findings. Clinical algorithms that use more stringent criteria are being developed and will require further validation (*31*,*43*).

## Evaluating Patients after Documentation of SARS Anywhere in the World

Once SARS activity has been documented anywhere in the world, the positive predictive value of even early clinical symptoms, while still low (*21*), is more acceptable if used in combination with an epidemiologic link to settings in which SARS has been documented. Therefore, in addition to evaluating all patients with unexplained pneumonia as described above, all patients with fever or respiratory symptoms should be screened for a history of exposure to persons with SARS, travel to areas where SARS transmission is suspected, or contact with ill persons with such a travel history.

In a community where transmission of SARS-CoV is widespread and many cases have no identifiable link to well-defined epidemiologic settings, a provisional diagnosis should be considered for any patient with fever or respiratory illness. The relationship between the clinical history, exposure history, and level of SARS activity in the surrounding community are summarized in Table 2.

The diagnosis of nosocomial SARS among patients hospitalized in either acute or long-term-care facilities may be particularly challenging, since many inpatients may have other reasons for having fever, respiratory symptoms, or pneumonia, and persons with other underlying illnesses may not have typical symptoms. Unrecognized nosocomial SARS was an important factor in spread of disease in the recent outbreaks described in Toronto, Singapore, and Taiwan (12*,**13**,**44*). Therefore, clinicians and public health professionals must stay particularly vigilant about evaluating fever and respiratory illnesses among inpatients if there have been recent SARS infections in the same facility (*44*).

## Management Decisions after Provisional Diagnosis

If a provisional diagnosis of SARS is made on the basis of the clinical and epidemiologic factors discussed, the patient should be managed according to existing guidance for SARS isolation precautions while evaluation and treatment proceed (*45*). The clinical evaluation should include, in addition to testing for SARS-CoV, laboratory testing for alternative diagnoses that could explain the illness. The patients should be isolated for the duration of the period of communicability or until convincing evidence against SARS is documented. Although the duration of communicability is not known, in the recent outbreak the isolation of patients until 10 days after their fever was gone and their respiratory symptoms were improving seemed an effective method to prevent additional transmission (*45*,*46*).

### Alternative Diagnoses

Documenting the presence of other diseases does not exclude the possibility of SARS because patients with SARS-CoV infection can be co-infected with other respiratory pathogens (*19*,*47*). If the presence of an alternative diagnosis is to be used as justification for discontinuing SARS-specific isolation precautions, the alternative diagnoses should be based only upon tests with high positive predictive value, and the clinical illness should be fully explainable by the diagnosis. The possibility of secondary infection should be considered if the diagnosis of bacterial pneumonia is confirmed, since bacterial pneumonia is a well-known complication of viral respiratory tract infection and may occur following SARS-CoV infection.

Particular care should be taken in completely attributing the illness to an alternative diagnosis if the epidemiologic link to others known to have SARS-CoV infection is strong, or if the patient is part of an epidemiologic cluster of similar illnesses. In the latter instance, confirming an alternative diagnosis among more than one person within the cluster may be used as evidence against SARS, particularly if the clinical findings are not typical of SARS (e.g., upper respiratory symptoms).

### Ruling out SARS

The only currently available laboratory method for excluding the diagnosis of SARS-CoV infection is to obtain a negative result on serologic testing of a convalescent-phase serum sample obtained >28 days after onset of symptoms. For patients without evidence of pneumonia at the initial evaluation, serial observations over time may be helpful in identifying those in whom isolation precautions can be safely discontinued (*21*). Resolution of symptoms and lack of development of radiographic evidence of pneumonia by the 2nd week of illness argue against the diagnosis of SARS. Some patients with mild illness may be missed when this approach is used, but if that is the case, they likely will not play an epidemiologically important role in transmission.

Patients with documented pneumonia who have been given the provisional diagnosis of SARS should be treated as if they have SARS-CoV infection, unless there is convincing evidence for an alternative diagnosis or new epidemiologic information excludes the possibility that the patient was exposed to SARS

## Importance of Communication

Because early recognition of SARS depends upon identifying the epidemiologic linkage to SARS-affected persons or places, clinicians must remain updated with current information regarding the locations of SARS activity in order to obtain the appropriate history from the patients with fever or respiratory illness. Mechanisms for rapid communication between clinicians and public health agencies must be in place so that physicians can be updated frequently as outbreaks evolve both locally and globally. Such lines of communication will also be important in helping public health agencies more rapidly identify emerging areas of activity (such as clusters of illness) through clinician reports of patients with risk factors for SARS.

Similarly, communication among health authorities in different jurisdictions in a region and among countries around the world will be essential to assess risk for exposure for travelers returning from those areas. Information on SARS can be obtained from CDC and WHO Web sites, among others (available from: URL: http://www.cdc.gov and URL: http://www.who.int).

## Conclusions

The framework that we have discussed for the early recognition of patients with SARS is based upon the knowledge and experience gathered during the recent worldwide outbreak, which suggests that clinical features alone cannot be used to conclusively distinguish SARS from other respiratory illnesses rapidly enough to inform early management decisions in a practical manner. Clinical features must be interpreted in the context of key epidemiologic risk factors, including epidemiologic linkage to other persons with pneumonia (i.e., clusters of cases of pneumonia clinically compatible with SARS), exposure to settings in which SARS activity is suspected or documented, and pneumonia among healthcare workers with direct patient care. Surveillance and additional research will be critical to help refine the epidemiologic, clinical, and laboratory features used to identify future infections with SARS, which will in turn help with the early detection and prevention of transmission of SARS-CoV infections.
